# Photonic Crystal Fiber SPR Liquid Sensor Based on Elliptical Detective Channel

**DOI:** 10.3390/mi12040408

**Published:** 2021-04-07

**Authors:** Xin Yan, Yao Wang, Tonglei Cheng, Shuguang Li

**Affiliations:** State Key Laboratory of Synthetical Automation for Process Industries, College of Information Science and Engineering, Northeastern University, Shenyang 110819, China; 1900745@stu.neu.edu.cn (Y.W.); chengtonglei@ise.neu.edu.cn (T.C.); lishuguang@ise.neu.edu.cn (S.L.)

**Keywords:** photonic crystal fiber, surface plasmon resonance effect, finite element method, liquid sensor, refractive index, sensitivity, linearity

## Abstract

This paper proposes a Photonic Crystal Fiber (PCF) refractive index sensor model based on the surface plasmon resonance effect. The proposed PCF model also uses the full vector finite element method to transfer the structure under the anisotropic Perfect Matching Layer (PML) boundary condition. Numerical calculations were carried out on the sensor characteristics. The calculation results show that the elliptical air hole on the left side of the PCF core is coated with a gold-nano film which serves as a Surface Plasmon Resonance (SPR) sensing channel to detect the refractive index of liquid materials. Compared with other structures, the resonant peak generated by the excited SPR effect from the elliptical sensing channel has a high sensitivity to the change of the refractive index of the liquid to be measured. With the help of this attribute, it is relatively easy to adjust the sensitivity. The refractive index range of this structure is within 1.43–1.49 and the sensitivity is up to 12,719.97 nm·RIU^−1^. The linearity is good; *R*^2^ = 0.99927, which is very suitable for liquid sensing.

## 1. Introduction

Photonic Crystal Fiber (PCF), when comparing it with the traditional fiber, shows more excellent optical characteristics due to its unique structure, such as non-stop single mode [1,2,3,4], low loss, high-nonlinearity [5,6], high-birefringence [7] and controllable dispersion [8], and large mode field area. According to different light transmission mechanisms, it can be divided into Total Internal Reflection Photonic Crystal Fiber (TIR-PCF) and Photonic Bandgap Photonic Crystal Fiber (PBGF). Comparing these two photonic crystal fibers, the TIR-PCF is simpler in principle and structure, and it is more suitable for optical fiber sensing. Surface Plasmon Resonance (SPR) is an optical phenomenon in which most of the energy of the incident light is absorbed by the plasma on the metal surface and the energy of the reflected light is drastically reduced. The SPR effect is sensitive to changes in the refractive index of the medium on the metal surface [9,10,11,12]. As we all know, the refractive index is the most basic optical parameter [13] and by testing the refractive index, various physical and optical properties, such as the concentration and purity of the substance, can be analyzed. Therefore, this feature is favorable to various types of optical fiber sensing and has broad application prospects and research value.

The PCF-SPR sensor uses the Surface Plasmon Polariton (SPP) mode and the core mode to produce the SPR effect when the phase-matching condition is met. This is because most of the energy in the core is coupled to the metal surface at this time and the energy in the core is rapidly reduced. This phenomenon is characterized by the confinement loss of the optical fiber, that is, the loss as the resonance wavelength increases sharply. The PCF-SPR sensor has also become a hot spot in various studies and research [14,15,16,17,18,19]. In 2006, Boris T. Kuhlmey et al. demonstrated that the confinement mechanism of PCFs with high refractive index-coated holes is wavelength dependent and exhibits plasma resonance in metal-coated PCFs. In the same year, P. Sazio of the University of Southampton enhanced the research of filling semiconductors and metal materials into PCF [20]. The Japanese research group Akira Nagasaki et al. also studied the sensing properties of gold nanowires filled in PCF and highlighted the reasons for the analysis in principle. Hassani also proposed two PCF-SPR sensor models with different structures and provided their application value [21,22].

The continuous growing research in this domain, to improve the dynamic detection range and sensitivity of SPR-PCF sensors, has become the most important research direction. In 2015, Ahmed A Rifat et al. proposed a solid-state core PCF sensor with graphene-silver deposition [23]. In 2016, Rahul Kumar Gangwar et al. proposed a surface plasmon resonance sensor based on D-type photonic crystal fiber for refractive index sensing [24]. The refractive index range is 1.43–1.46, and the average sensitivity is 7700 nm·RIU^−1^. In 2017, Guowen An et al. proposed a D-type photonic crystal fiber sensor based on surface plasma resonance [25]. The results show that the refractive index range is 1.33–1.38, and the maximum sensitivity is 10,493 nm·RIU^−1^. Liu et al. put gold nanowires into PCF in 2018, and the detection range of this sensor is 1.33–1.38, and the highest sensitivity is 4111 nm·RIU^−1^ [26]. In the same year, Vigneswaran et al. reported a PCF-based salinity sensor and found a maximum salinity sensitivity of 5675 nm·RIU^−1^ [27].

Based on such sensors, the current dynamic detection range is small and the sensitivity is low and not highly adjustable. This paper proposes a PCF refractive index sensor based on the SPR effect which introduces an asymmetric cladding structure and an elliptical detection channel. This method widens the dynamic detection range between 1.43–1.49 and a sensitivity of up to 12,719.97 nm·RIU^−1^, with good linearity which is suitable for liquid sensing with the high refractive index.

## 2. Structural and Theoretical Modeling

**Figure 1:**
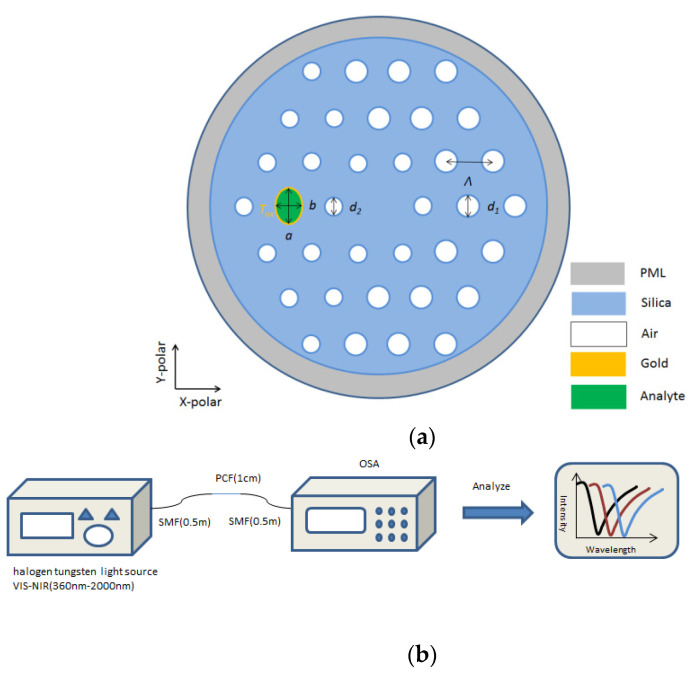
(**a**) Across-sectional schematic structure of the Surface Plasmon Resonance Photonic Crystal Fiber (SPR-PCF) refractive index sensor. (**b**) Illustration of experimental setup of the proposed sensor.

The cross-sectional structure for our proposed sensor is shown in Figure 1a. The cladding layer is formed by an asymmetric regular hexagonal air hole arranged with a lattice constant *Λ*. The diameter of the large and small air holes is *d*_1_ and *d*_2_ and the refractive index of air is *n_air_* = 1. These airholes help to lessen the confinement loss effectively and also help to raise up the birefringence slightly. An elliptical sensor channel coated with gold is introduced on the left side of the core to place the liquid to be detected. The gold layer coating thickness of the elliptical channel hole wall is *T_ox_*. One of the most important aims of this paper is to analyze the sensing performance. After watchful study, the ideal airhole parameters are *Λ* = 2 μm, *d*_1_ *=* 0.8*Λ* and *d*_2_ *=* 0.6*Λ*. When gold is used as the plasmonic material, we have kept the thickness of gold at 40 nm. The elliptical detection channel initial length is *b*_0_ = 0.8*Λ,* the ellipticity is *e* = *a/b*, and the initial is set at *e*_0_ = 0.7. Finally, the refractive index of the liquid to be detected is *N_a_*, to set the initial structural parameters.

The substrate and core materials of PCF are silicon dioxide, and the dispersion of the material can be obtained from the Sellmeier Equation (1):

(1) $$n^{2}\;=\;1\;+\;\overset{3}{\underset{i\;=\;1}{\sum }}\frac{B_{i}\Lambda^{2}}{\Lambda^{2}{-C}_{i}{}^{2}}$$

where *n* is the wavelength-dependent refractive index of fused quartz; *Λ* is the input wavelength; and *B*_1_ = 0.6961663, *B*_2_ = 0.4079426, *B*_3_ = 0.8974764, *C*_1_ = 0.0684043 µm, *C*_2_ = 0.1162414 µm, *C*_3_ = 9.896161 µm.

Gold is used as the plasmonic metal for its stability as a chemical element. The dielectric constant of gold can be derived using the Drude‒Lorentz formula:

(2) $$\varepsilon_{\;\left(\omega \right)}\;=\;\;\varepsilon_{1}\;+\;i\varepsilon_{2}\;=\;\;\varepsilon_{\infty }\;-\;\frac{\omega_{p}^{2}}{\omega \;\left(\omega +i\omega_{c}\right)}$$

where $\varepsilon_{1}$ is the value of the real part of the metal-dielectric constant; $\varepsilon_{2}$ is the value of the imaginary part of metal-dielectric constant; and ω is the angular frequency of the incident wave. The value of a metal-dielectric constant $\omega_{\infty }$ is 9.84. The plasma $\omega_{p}$ frequency was 1.36 × 10^16^ rad/s. The damping frequency $\omega_{c}$ is 1.45 × 10^14^ rad/s.

All the simulations in this paper are based on COMSOL software. The fiber structure of this paper is analyzed in the wave optics module. The effective refractive index and loss of the core mode and SPP mode at each wavelength is calculated to make further study. Figure 1b shows an illustration of the experimental setup of the proposed sensor. The liquid to be detected is filled in the detection channel, and the liquid refractive index is changed due to the change of the external environment. The single-mode fiber (SMF) is fused at both ends of the photonic crystal fiber (PCF). We consider 0.5 m SMF and 1 cm PCF should be used, as the confinement loss is 3–4 dB/cm. If it is too long, unnecessary loss will be increased and the results will be affected, and the light emitted by the light source is output after passing through the PCF. The light source we chose is Hogen Tungsten Light Sourcevis-Nir (360–2000 nm), which is a broad band light source that can emit 360–2000 nm light for us to use. Then, this is received and analyzed using an optical spectrum analyzer (OSA). The OSA directly analyzes the intensity of the emitted light, under the premise of ensuring that the incident light is parallel to the PCF. In OSA analysis, there is a trough in the intensity of the emitted light. When the liquid changes significantly, the trough moves accordingly. We use this phenomenon to determine the resonance wavelength at which the SPR effect occurs. In our design, the change of the wave trough is consistent with the change of the loss peak in the paper, that is, with the increase of the refractive index, the wave trough will move to the long wave direction.

Confinement loss is the light confinement ability within the core region. Due to confinement loss, the light signal will be weakened as it propagates distance through the fiber. The confinement loss (*CL*) formula is stated as:

(3) $$CL=8.686*k_{0}*Im\left(n_{eff}\right)*10^{4}\;\left(\mathrm{dB}/\mathrm{cm}\right)$$

where 8.686 is confinement loss coefficient (4.343) multiplied by attenuation coefficient *α* (when the confinement loss unit is dB/cm, *α* = 2/cm), $k_{0}=\frac{2\pi }{\lambda }$ is the wave number in the free space, *Λ* is the operating wavelength, $Im\;\left(n_{eff}\right)$ is the imaginary portion of effective index and 10^4^ is converted from units [28]. The confinement loss is proportional to the imaginary portion of the effective refractive index. The larger the loss value, the stronger the SPR effect at that point.

## 3. Simulation Results and Analysis

The structure proposed in this paper is asymmetric, therefore, it is going to introduce birefringence. This will cause the incident light to produce light in two different transmission directions, namely *x*-polarized light and *y*-polarized light, during core propagating. The first thing to discuss is the intensity of the core modes in different polarization directions when they resonate with the metal surface plasmon.

**Figure 2:**
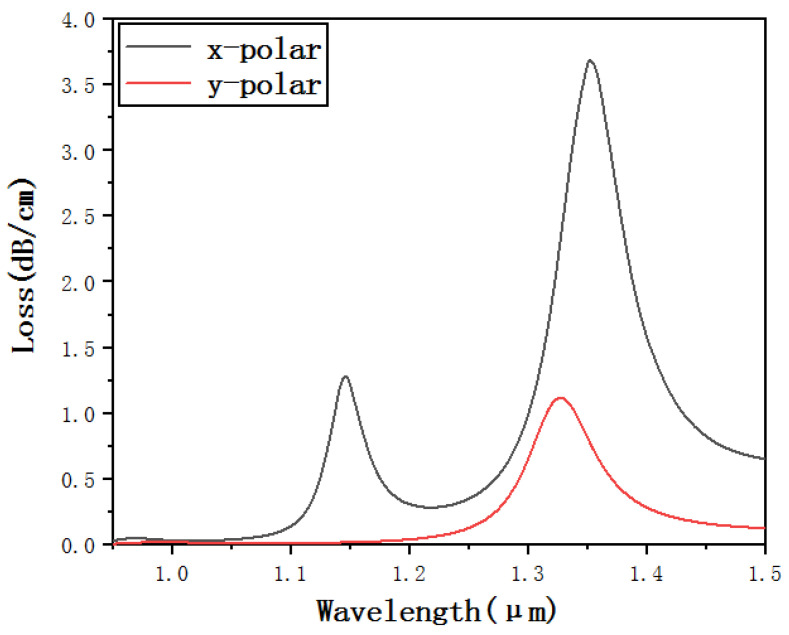
Confinement loss curve of the core mode of *x*- and *y*-polarization states when *N_a_* = 1.45.

Figure 2 is the loss curve of the core mode of *x*-polarization state and the core mode of *y*-polarization state when the refractive index of the liquid is *N_a_* = 1.45 and the studied waveband is 0.9–1.5 μm. It can be seen from Figure 2 that both the *x*- and *y*-polarized core modes can resonate with the plasmon (SPP) mode. It is not difficult to conclude from the magnitude relationship of the confinement loss that the SPR effect excited by the coupling of the core mode and the SPP mode in the *x*-polarization state is stronger than the SPR effect excited by the coupling of the core mode and the SPP mode in the *y*-polarization state. This is because when the light in the fiber core is coupled with the plasmon on the metal surface, the electromagnetic field on the metal surface will be excited. In this way, the plasma on the metal surface can oscillate to the *x*-polarization state and the *y*-polarization state at the same time, that is, the SPR effect will occur in both the *x*-polarization state and the *y*-polarization state. However, since the gold-plated film is in the *x*-polarization state, it will make the plasma more likely to oscillate in the *x*-polarization state than in the *y*-polarization state. As a result, the coupling efficiency of the *x*-polarization state is much higher than that of *y*-polarization state. Therefore, the SPR effect produced by coupling the core mode of the *x*-polarization state with the SPP mode is stronger and easier to distinguish and is more suitable for sensing. This article is based on the analysis of the sensing characteristics obtained by the SPR effect, which is generated by the excitation of the core mode and the SPP mode in *x*-polarization state.

**Figure 3:**
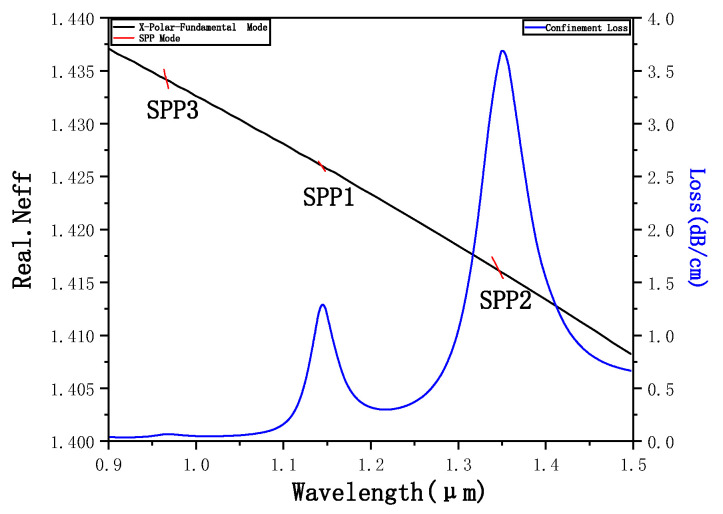
Dispersion and confinement loss curve of the fundamental mode and the Surface Plasmon Polariton (SPP) mode in the *x*-polarization state when *N_a_* = 1.45.

Figure 3 shows the dispersion curve of the fundamental mode and the SPP mode in the *x*-polarization state, both the confinement loss curve of the fundamental mode in the *x*-polarization state when *N_a_* = 1.45. It can be seen from the figure that within the range of 0.9–1.5 μm, the confinement loss of the fundamental mode in the *x*-polarization state has three different peaks. According to the previously described theory, there are three SPR effects of the fundamental mode in the *x*-polarization state, resulting in three resonance loss peaks. It can be seen from the figure that each resonance loss peak is a point where the real portion of the effective refractive index of the fundamental mode is equal to the real portion of the effective refractive index of the SPP mode in the *x*-polarization state. This is because the wave vectors of the fundamental mode and the SPP mode are equal at this time, and the phase-matching condition is met. The SPR effect occurs so that the energy in the core is largely absorbed by the plasma and the loss reaches the peak.

**Figure 4:**
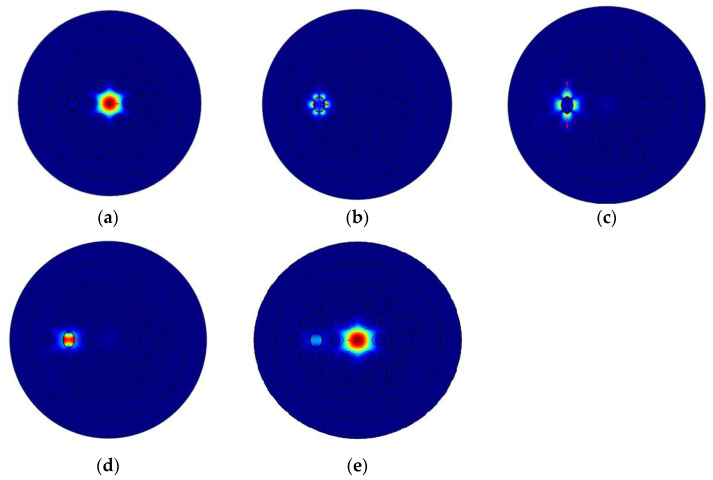
(**a**) The core-guided mode, (**b**) the SPP3 mode, (**c**) the SPP2 mode, (**d**) the SPP1 mode and the (**e**) optical field distribution in phase matching (*N_a_* = 1.45)**.**

Figure 4a shows the optical field of the core-guided mode and it mainly distributes in the core. Figure 4b–d shows the optical field distribution of the three SPP modes and it mainly distributes on the surface of gold film. Figure 4e shows the optical field of the core-guided mode and the SPP mode at the resonance wavelength. We can see that there is a strong coupling between the two modes, and the optical field distributes both on the surface of the gold film and in the core. This confirms the occurrence of the phase-matching condition.

**Figure 5:**
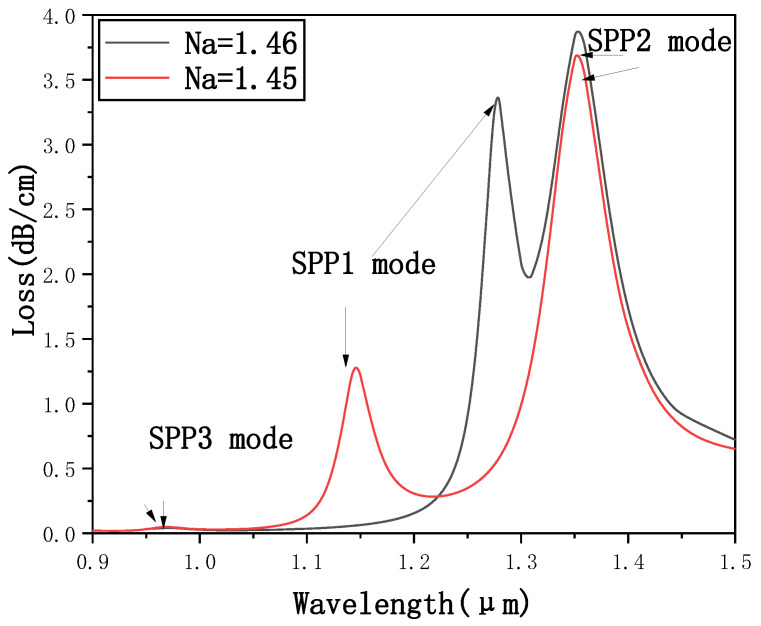
Confinement loss curves of *x*-polarization fundamental mode when *N_a_* = 1.45 and *N_a_* = 1.46.

Figure 5 shows the confinement loss curve of the core mode in the *x*-polarization state when *N_a_* = 1.45 and *N_a_* = 1.46. It is not difficult to see that the SPR effect excited by the fundamental mode and SPP3 mode is the weakest and it is not easy to distinguish. The resonance wavelength does not change significantly with the refractive index of the liquid to be detected and the sensitivity is low. Therefore, the SPR effect excited by the SPP3 mode is not suitable for liquid sensing. The SPR effect motivated by the fundamental mode and the SPP2 mode is the strongest. Although it is easy to distinguish, the resonance wavelength does not change significantly with the refractive index of the liquid to be tested. The sensitivity is low and the SPR effect motivated by the SPP2 mode is not suitable for liquid sensing. The SPR effect stimulated by the fundamental mode and SPP1 mode is relatively strong and relatively easy to distinguish, and the resonance wavelength changes significantly with the refractive index of the liquid to be tested. This is because most of the light field is concentrated in the elliptical channel hole. When the refractive index of the liquid to be detected changes, the range of the resonance wavelength varying with the refractive index of the liquid to be detected is the largest and the sensitivity is the best. In summary, the SPR effect excited by the SPP1 mode is the most suitable for liquid sensing. The following research in this paper is based on the resonance wavelength of the SPR effect excited by the coupling of the SPP1 and fundamental modes with the refractive index of the liquid to be detected.

**Figure 6:**
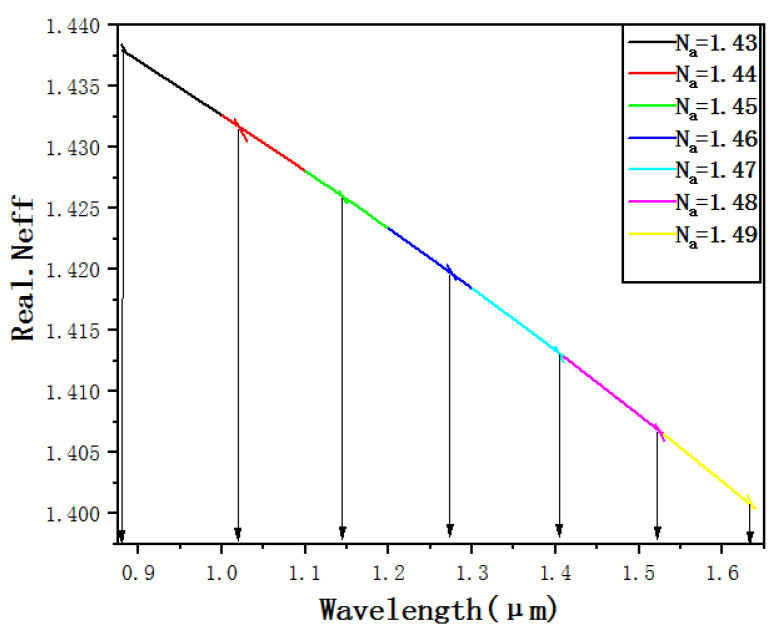
The dispersion curve and the resonance wavelength when *N_a_* range is 1.43–1.49.

Figure 6 shows the dispersion curve and the resonance wavelength when the refractive index *N_a_* of the liquid to be detected is within the range of 1.43 to 1.49. It can be seen from the figure that when *N_a_* changes from 1.43 to 1.49, the resonance wavelengths corresponding to the SPR effect motivated by the SPP1 mode are 885 nm, 1019 nm, 1146 nm, 1278 nm, 1404 nm, 1523 nm and 1636 nm, according to the wavelength sensitivity formula:

(4) $$S_{\;\left(\lambda \right)}=\;\Delta \lambda_{rw}\;\left(N_{a}\right)/\Delta N_{a}$$

The average sensitivity of each segment can be calculated as 13,400 nm·RIU^−1^, 12,700 nm·RIU^−1^, 13,200 nm·RIU^−1^, 12,600 nm·RIU^−1^, 11,900 nm·RIU^−1^ and 11,300 nm·RIU^−1^.

**Figure 7:**
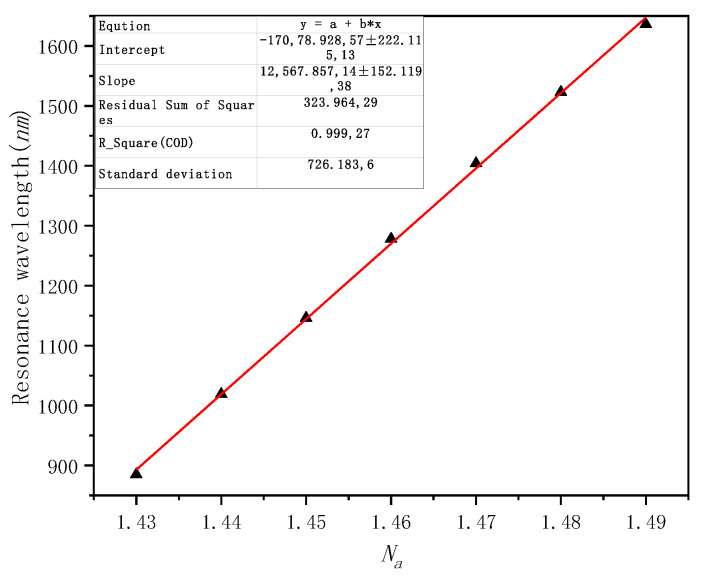
Wavelength sensitivity fitting curve.

The values of the refractive index of different liquids are detected and their resonance wavelength into the fitting formula are obtained along with the fitting curve, as shown in Figure 7. Further fitting calculations demonstrate the average sensitivity of the structure, that is, 12,567.85 nm·RIU^−1^, and the maximum sensitivity is 12,719.97 nm·RIU^−1^. *R^2^* = 0.99927, which shows that the linear fit is good.

Wavelength resolution is one of the crucial sensing parameters which shows how a minute change in refractive index can be detected by the sensor. A lower value of wavelength resolution gives rise to a better capability of detecting minute RI change. Wavelength resolution can be calculated as

(5) $$R\;\left(RIU\right)\;=\frac{\Delta N_{a}*\Delta \lambda_{minimum}}{\Delta \lambda_{rw}}$$

where Δ$\lambda_{rw}$ is the resonance wavelength difference, Δ$N_{a}$ is the change in refractive index of the liquid to be detected and Δ$\lambda_{minimum}$ is generally assumed to be 0.1 nm. However, our proposed PCF shows minimum resolution of 7.46 × 10**^−^**^6^ RIU.

**Figure 8:**
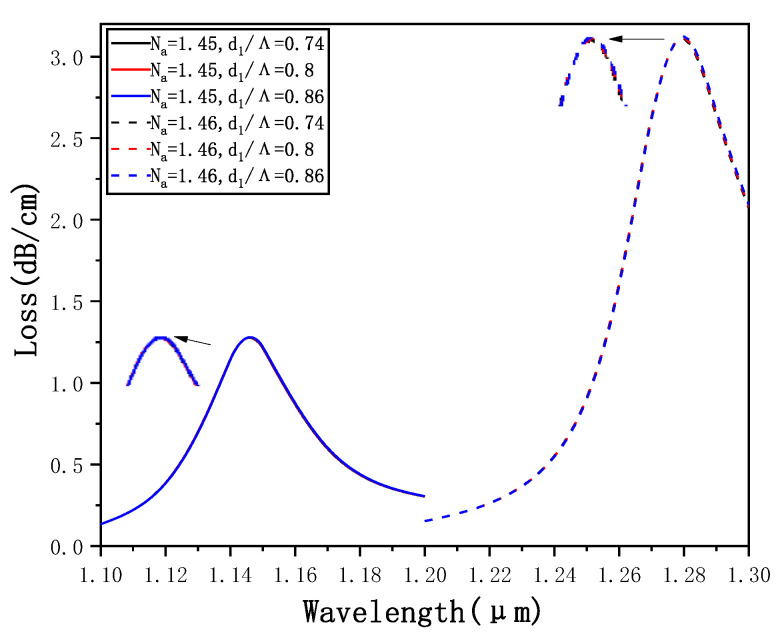
Confinement loss curve of *d*_1_ = 0.74*Λ*, *d*_1_ = 0.8*Λ* and *d*_1_ = 0.86*Λ* when *N_a_* = 1.45 and *N_a_* = 1.46.

Figure 8 shows a confinement loss curve of *d_1_* = 0.74*Λ*, *d*_1_ = 0.8*Λ* and *d*_1_ = 0.86*Λ* when *N_a_* = 1.45 and *N_a_* =1.46. A longitudinal comparison shows that, when *N_a_* remains unchanged, only *d*_1_ increases, and the resonance wavelength and the loss peak show no significant change. A horizontal comparison shows that when Na changes from 1.45 to 1.46, the resonance wavelength and the loss peak are going to increase gradually for the same d1 size. However, we can find that the sensitivity has no obvious change. Theoretically, the reason is that the large air holes are arranged in the outer layer of the entire cladding structure and the air holes around the core are composed of small air holes. When *d*_1_ is changed only, the coupling between the core mode and SPP1 mode and sensitivity is not going to be affected.

**Figure 9:**
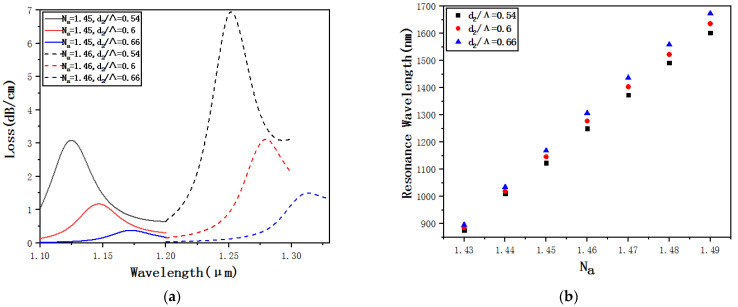
(**a**) Confinement loss curves of *d*_2_ = 0.54*Λ*, *d*_2_ = 0.6*Λ* and *d*_2_ = 0.66*Λ* when *N_a_* = 1.45 and *N_a_* = 1.46. (**b**) Resonance wavelengths of *d*_2_ = 0.54*Λ*, *d*_2_ = 0.6*Λ* and *d*_2_ = 0.66*Λ* when *N_a_* changes from 1.43 to 1.49.

Figure 9a shows the confinement loss curves of *d*_2_ = 0.54*Λ*, *d*_2_ = 0.6*Λ* and *d*_2_ = 0.66*Λ* when *N_a_* = 1.45 and *N_a_* = 1.46. The longitudinal comparison shows that, when *N_a_* remains unchanged, only *d*_2_ increases, and the resonance wavelength is going to gradually increase. The loss peak is going to gradually decrease. Theoretically, the reason is that the small air holes are not only arranged in the outer layer of the cladding structure but also arranged around the core. When *d*_2_ increases, it gradually prevents the fiber core from coupling with the plasma on the surface of the elliptical detection channel, and the SPR effect gradually weakens and the loss peak gradually decreases. A horizontal comparison shows that when *N_a_* changes from 1.45 to 1.46, for the same *d*_2_ size, both the resonance wavelength and the loss peak are going to gradually increase. Figure 9b shows the resonance wavelengths of *d*_2_ = 0.54*Λ*, *d*_2_ = 0.6*Λ* and *d*_2_ = 0.66*Λ* when *N_a_* changes from 1.43 to 1.49. Through fitting calculations, we can get an average sensitivity of 12*,*125 nm·RIU^−1^ when *d*_2_ = 0.54*Λ*, 12*,*567 nm·RIU^−1^when *d*_2_ = 0.6*Λ*, and when *d*_2_ = 0.66*Λ*, the average sensitivity is 13*,*035 nm·RIU^−1^. The conclusion is that as *d*_2_ increases, the sensitivity increases to a certain extent and the size of d_2_ affects the sensitivity. To ensure the sensitivity, d_2_ should be slightly larger.

**Figure 10:**
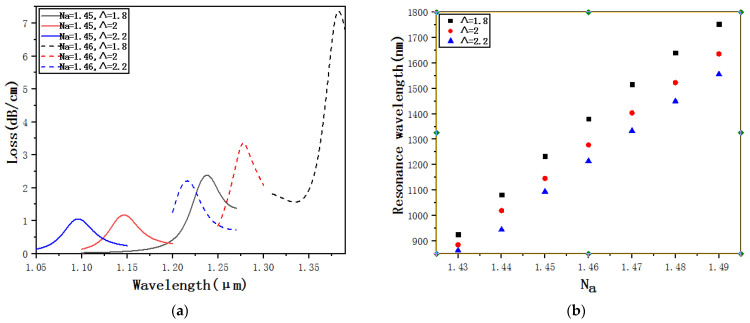
(**a**) Confinement loss curves of *Λ* = 1.8, *Λ* = 2 and *Λ* = 2.2 when *N_a_* = 1.45 and *N_a_* = 1.46. (**b**) Resonance wavelengths of *Λ* = 1.8, *Λ* = 2 and *Λ* = 2.2 when *N_a_* changes from 1.43 to 1.49.

Figure 10a shows the confinement loss curves of *Λ* = 1.8, *Λ* = 2 and *Λ* = 2.2 when *N_a_* = 1.45 and *N_a_* = 1.46. A longitudinal comparison shows that when *N_a_* remains unchanged, only *Λ* increases, and both the resonance wavelength and the loss peak are going to gradually decrease. Theoretically, the reason is that when *Λ* increases, the distance between the core and the ellipse detection channel gradually increases, making it much more difficult to couple the core and the plasma on the gold coating surface. The SPR effect gradually weakens and the loss peak gradually decreases. Horizontal comparison for the same size of *Λ* shows that when *N_a_* changes from 1.45 to 1.46, both the resonance wavelength and the loss peak are going to gradually increase. Figure 10b shows the resonance wavelengths of *Λ* = 1.8, *Λ* = 2 and *Λ* = 2.2 when *N_a_* changes from 1.43 to 1.49. Through fitting calculations, the average sensitivity is 13,875 nm·RIU^−1^ when *Λ* = 1.8, the average sensitivity is 12,567 nm·RIU^−1^ when *Λ* = 2, and the average sensitivity is 11,903 nm·RIU^−1^ when *Λ* = 2.2. That is, as *Λ* increases, the sensitivity decreases obviously. Thus, the size of *Λ* affects the sensitivity. To ensure the sensitivity, *Λ* should be slightly smaller.

**Figure 11:**
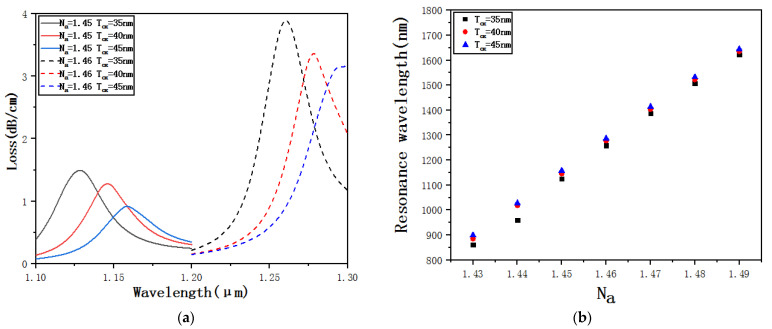
(**a**) Confinement loss curves of *T_OX_* = 35 nm, *T_OX_* =40 nm and *T_OX_* =45 nm when *N_a_* = 1.45 and *N_a_* = 1.46. (**b**) Resonance wavelengths of *T_OX_* = 35 nm, *T_OX_* =40 nm and *T_OX_* =45 nm when *N_a_* changes from 1.43 to 1.49.

Figure 11a shows the confinement loss curves of *T_OX_* = 35 nm, *T_OX_* = 40 nm and *T_OX_* = 45 nm when *N_a_* = 1.45 and *N_a_* = 1.46. A longitudinal comparison shows that when *N_a_* remains unchanged, only *T_OX_* increases, and the resonance wavelength is going to gradually increase. The loss peak is going to gradually decrease. Theoretically, the reason is that when *T_OX_* increases, due to the increase in the thickness of the gold-plated film, the distance between the core and the elliptical detection channel is indirectly increased and the coupling strength is weakened, which makes the SPR effect gradually weaken and the loss peak gradually decrease. A horizontal comparison shows that for the same size of *T_OX_*, when *N_a_* changes from 1.45 to 1.46, both the resonance wavelength and the loss peak are going to gradually increase. Figure 11b shows the resonance wavelengths of *T_OX_* = 35 nm, *T_OX_* = 40 nm and *T_OX_* = 45 nm when *N_a_* changes from 1.43 to 1.49. Through fitting calculations, we can get that the average sensitivity when *T_OX_* = 35 nm is 13,014 nm·RIU^−1^, the average sensitivity when *T_OX_* = 40 nm is 12,567 nm·RIU^−1^, and the average sensitivity when *T_OX_* = 45 nm is 12,503 nm·RIU^−1^. That is, as the *T_OX_* increases, the sensitivity gradually decreases and the *T_OX_* size affects the sensitivity. To ensure the sensitivity, *T_OX_* should be slightly smaller.

**Figure 12:**
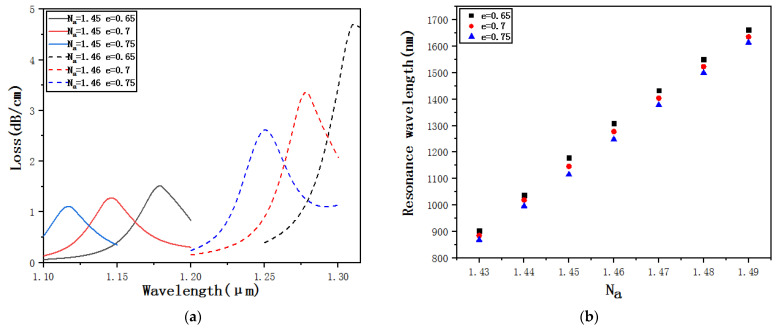
(**a**) Confinement loss curves of *e* = 0.65, *e* = 0.7 and *e* = 0.75 when *N_a_* = 1.45 and *N_a_* = 1.46. (**b**) Resonance wavelengths of *e* = 0.65, *e* = 0.7 and *e* = 0.75 when *N_a_* changes from 1.43 to 1.49.

Figure 12a shows the confinement loss curves of *e* = 0.65, *e* = 0.7 and *e* = 0.75 when *N_a_* = 1.45 and *N_a_* = 1.46. *e* is changed by changing the length *b* but keeping the length *a* unchanged. A longitudinal comparison shows that when *N_a_* remains unchanged, only *e* increases, and both the resonance wavelength and the loss peak are going to gradually decrease. Theoretically, the reason is that when *e* increases, the length *b* decreases, resulting in the area of the elliptical detection channel decreasing, which weakens the coupling between the core, and the SPR effect gradually weakens and the loss peak decreases. The horizontal comparison shows that when *N_a_* changes from 1.45 to 1.46, for the same size of *e*, both the resonance wavelength and the loss peak are going to gradually increase. Figure 12b shows the resonance wavelengths of *e* = 0.65, *e* = 0.7 and *e* = 0.75 when *N_a_* changes from 1.43 to 1.49. Through fitting calculations, we can get that the average sensitivity when *e* = 0.65 is 12,710 nm·RIU^−1^, when *e* = 0.7 is 12,567 nm·RIU^−1^, and when *e* = 0.75 is 12,532 nm·RIU^−1^. It can be found that as *e* increases, the sensitivity gradually decreases, that is, the size of *e* affects the sensitivity, but the degree of the influence is very low.

## 4. Conclusions

Compared with other sensors, our proposed PCF sensor has many advantages. Based on the regular hexagon structure, there are two kinds of air holes, *d*_1_ and *d*_2_. Although the size is different, the arrangement is regular. Elliptical detection hole can also be obtained by controlling the temperature and air pressure during optical fiber drawing, so it is relatively simple. For metal-coated film, we only coat a gold film in the elliptical detection hole to get a better result. Compared with the traditional round hole, the elliptical hole is easier to make the SPR effect occur, so the change of the liquid in the elliptical detection hole is more sensitive to the resonance and easier to distinguish. Our refractive index range is relatively wide, the sensitivity is also high, the linear correlation is closer to 1 and the linear correlation is better. Thus, it has a certain value in the future for biological sensing and chemical sensing. For example, we consider filling magnetic fluid materials to make a sensor relating to magnetic field and temperature, which is feasible. As for some details about the practical design, we will use the capillary stacking method to make the prefabricated rod. According to the cross-sectional structure for our PCF, we need 36 glass tubes, including 17 thick-walled glass tubes of 8 mm inside diameter and 20 mm outside diameter (8 mm/20 mm), 18 thin-walled glass tubes (12 mm/20 mm) and 2–3 thin-walled glass tubes (18 mm/20 mm), which are put into a (14 mm/20 mm) glass tube. We put the prefabricated rod in the special fiber wire drawing tower of the high temperature graphite furnace and heat it. We control the temperature at 1800 °C and control the air pressure at 5 kpa. In order to adjust the size of the air holes and obtain the elliptical air hole, through controlling the pressure, the thin walls of the air holes (18 mm/20 mm) are squeezed and broken to become the ellipse. In this way, the optical fiber can be made successfully. Then, we consider using UV glue to seal air holes other than elliptical air holes under the light microscope. We can also consider the use of arc discharge, as long as the discharge intensity and discharge time is well controlled; it can also make other air holes except elliptical air holes collapse. We can use chemical vapor deposition (CVD) to coat the wall of the elliptical air hole with gold. We can also use the simple principle of capillary siphon, so that the liquid smoothly fills into the ellipse detection hole. However, we use an arc discharge-type welding machine, but the welding time is very short. In the “ms” magnitude, because of such a short time, the liquid will not have enough time to evaporate, so the filling liquid will basically be retained. That is everything about the practical design; we will conduct follow-up research and discussion on these issues.

In this paper, a photonic crystal fiber liquid refractive index sensor is proposed, designed and simulated which is based on the SPR effect. The refractive index of the liquid to be detected is 1.43–1.49. The average sensitivity is 12,567.85 nm·RIU^−1^ and the maximum sensitivity is 12,719.97 nm·RIU^−1^. This has a certain improvement in dynamic detection range and sensitivity with a good linear fit. The structure is sensitive to several parameters of the optical fiber, and a better dynamic detection range and sensitivity can be obtained by changing the parameters of the optical fiber. It provides a reference for studying the SPR-PCF refractive index sensor.
